# Tracheobronchomegaly (Mounier-Kuhn Syndrome) with CT and bronchoscopic correlation: A case report

**DOI:** 10.1016/j.radcr.2022.06.077

**Published:** 2022-07-29

**Authors:** Aboubekr Imzil, Fatiha Bounoua, Hicham Naji Amrani, Houda Moubachir, Hind Serhane

**Affiliations:** a Pneumology Department, Faculty of Medicine and Pharmacy of Agadir, Souss-Massa University Hospital, Ibn Zohr University, Agadir, Morocco; b Pneumology Department, Faculty of Medicine and Pharmacy of Agadir, Oued Eddahab Military Hospital, Ibn Zohr University, Agadir, Morocco

**Keywords:** Tracheobronchomegaly, Mounier-Kuhn syndrome, Recurrent respiratory infections, The therapeutic management

## Abstract

Tracheobronchomegaly, or Mounier-Kuhn syndrome, is a clinical and radiological entity characterized by marked dilatation of the trachea and bronchi as a result of severe atrophy of the elastic fibers, with thinning of the muscularis, and the formation of diverticula between the cartilaginous rings. The etiopathogenesis is uncertain and may be congenital or acquired. The clinical signs are not specific and are frequently revealed by recurrent respiratory infections and chronic cough. The diagnosis of Mounier-Kuhn syndrome is based on well-documented measurements of the trachea and main bronchi performed on a chest computed tomography scan. The management of patients is based on symptomatic treatment and may require, in severe cases, the use of endoscopic treatment by stent placement or surgical tracheobronchoplasty. We present a case of a 59yearold patient with recurrent respiratory infections that required several hospitalizations. Diagnosed with Mounier Kuhn syndrome, the thoracic computed tomography scan demonstrated a dilated trachea until the bifurcation and focal points of bronchial dilatation. Bronchoscopic examination showed a dilated and deformed trachea with the presence of diverticula on the tracheal anterior wall. The diameter of the trachea was reduced by more than 50% during expiration and coughing. For this reason, Mounier-Kuhn syndrome should be considered in cases of recurrent respiratory infection or persistent respiratory symptoms.

## Background

Tracheobronchomegaly, also known as Mounier-Kuhn syndrome, is a rare disease whose congenital or acquired origin is still being discussed. It is characterized by an important dilatation of the trachea and the proximal bronchi, responsible for recurrent respiratory infections [1]. Clinical signs are not very specific [2]. Radiological diagnosis is often easy on chest X-ray and computed tomography (CT) scan based on the measurement of the diameter or surface of the trachea and stem bronchi [3].

The objective of this report is to increase knowledge about this chronic disease, ensuring early diagnosis, and appropriate management.

## Case report

A 59-year-old man, a plumber and electrician, with a history of type II diabetes, depression, and recurrent respiratory infections (3 episodes per year), was revealed by a productive cough associated with infectious syndrome. The patient had an antecedent of hospitalization in a pneumology department for a respiratory distress with minor hemoptysis. He is currently consulting for exertional dyspnea that has progressively aggravated and is associated with cough productive of white sputum. A Covid-19 infection was excluded in the patient who had received 3 doses of the Covid-19 vaccine.

A physical examination revealed a respiratory rate of 20 c/m. Oxygen saturation was 92% increased to 98% less than 2 liters of oxygen. He had bilateral bronchial rales on auscultation and excess skin on the upper and lower lip.

**Fig. 1:**
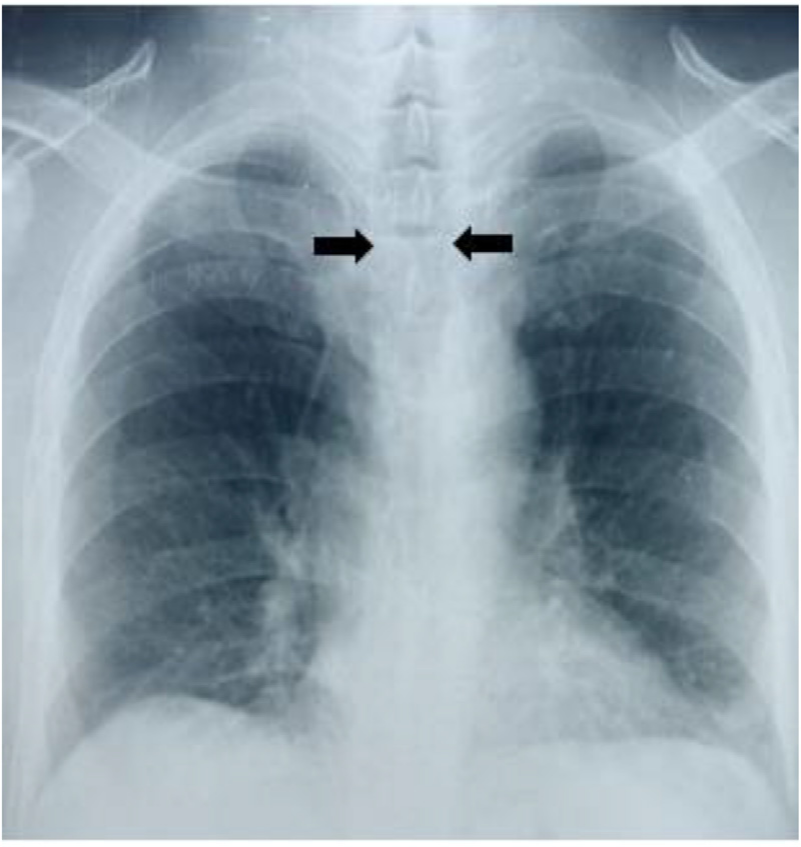
Frontal chest X-ray: marked enlargement of tracheal clarity with thoracic distension.

The chest X-ray showed marked enlargement of the tracheal clearness, thoracic distension with widening of the intercostal spaces (Fig. 1).

**Fig. 2:**
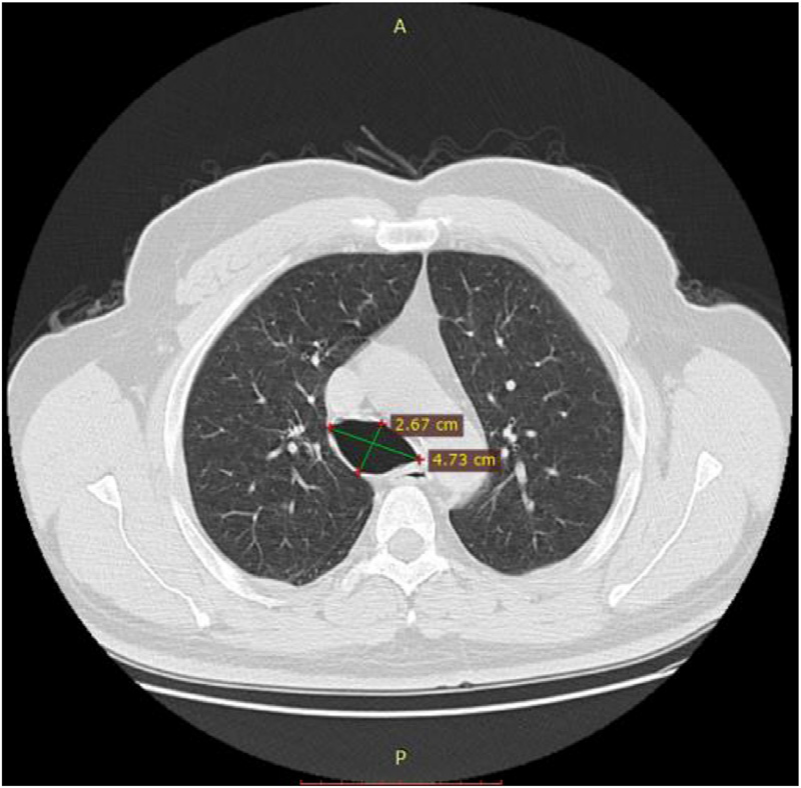
Thoracic CT scan: axial section through the parenchymal window passing through the trachea. The transverse diameter of the trachea is 47.3 mm; the sagittal diameter is 26.7 mm.

**Fig. 3:**
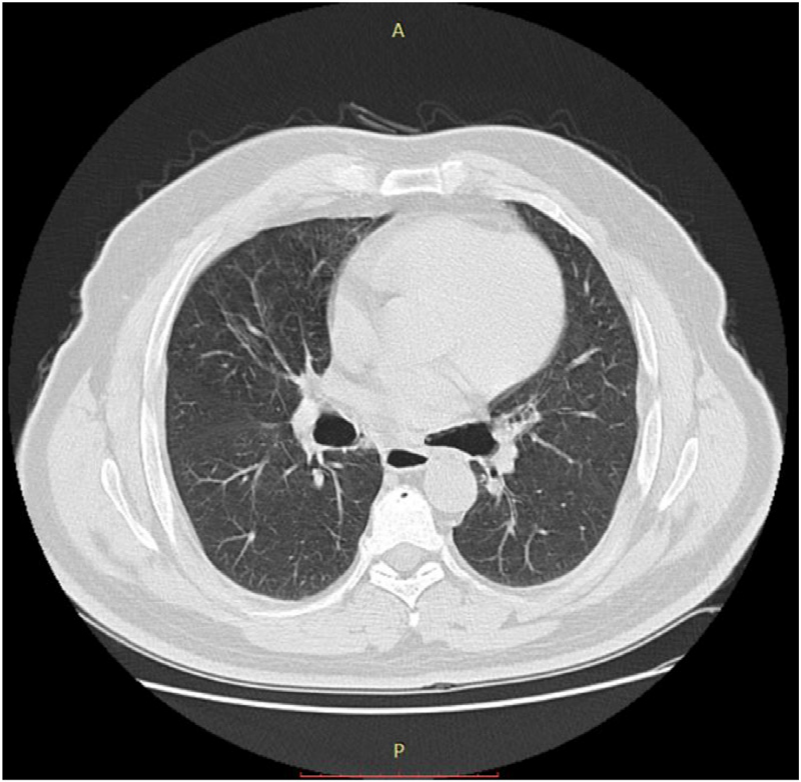
Thoracic CT scan: axial sections through the parenchymal window showing dilatation of the right and left main bronchi.

**Fig. 4:**
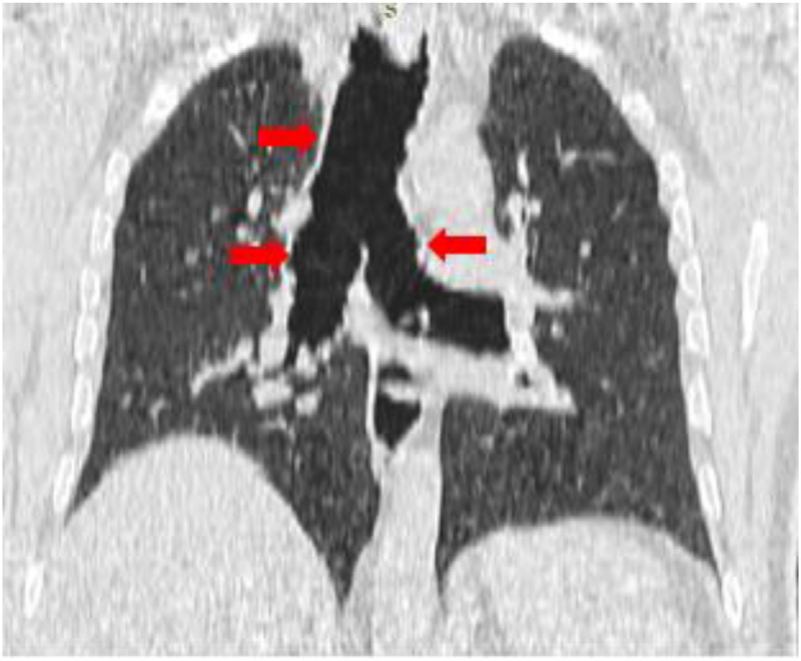
Chest CT scan: coronal reconstruction in a plane passing through the trachea and main bronchi showing tracheobronchomegaly.

A thoracic CT scan with helical acquisition over the chest in spontaneous contrast was performed (Fig. 2, Fig. 3 and 4):

• A dilated trachea until the bifurcation. The anteroposterior tracheal diameter was 26.7 mm and the transverse diameter was 47.3 mm.
• Cylindrical moniliform bronchial dilatations in the middle lobar region, the underlying lobe are reduced.

Cylindrical bronchial dilatations in the lower lobar without active parenchymal lesions, lytic bone lesions, or suspicious condensation

**Fig. 5:**
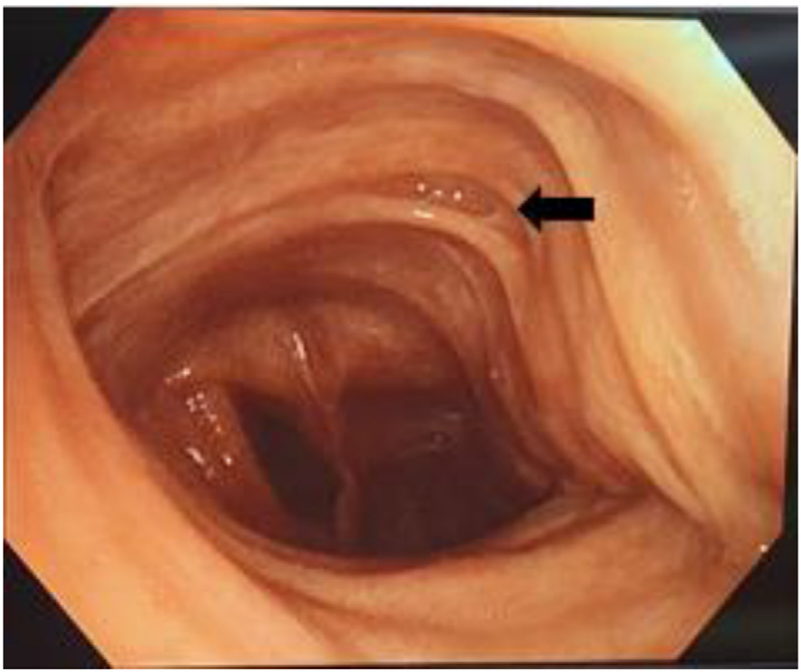
Bronchoscopy: massively dilated trachea with a diverticular formation.

The bronchoscopy showed a dilated and deformed trachea with spreading of the membranous part at the expense of the tracheal cartilage and the presence of diverticula on the tracheal anterior wall. The diameter of the trachea was reduced by more than 50% during expiration and coughing, with the inflammatory appearance and presence of mucopurulent secretions in the right and left bronchial tree (Fig. 5).

The patient's cardiac evaluation did not show any abnormality. Arterial gasometry revealed normocapnia with mild hypoxemia at 75 mm Hg.

During hospitalization, the patient was treated with, oxygen and antibiotic therapies; inhaled corticosteroid associated with a long-acting bronchodilator; respiratory physiotherapy; influenza, and pneumococcal vaccination were recommended for the patient after leaving the hospital.

## Discussion

Congenital tracheobronchomegaly, or Mounier-Kuhn syndrome, is a rare clinical and radiological entity, which was described firstly by Mounier-Kuhnin 1932 [4]. The syndrome is characterized by a significant dilatation of the trachea and bronchi [4], [5], [6], [7], [8], [9], [10], [11]. The majority of the cases are presented in the third or fourth decade with recurrent respiratory infections. It is more common in men [12].

Tracheobronchomegaly is characterized by a severe atrophy of the longitudinal elastic fibers, with a thinning of the muscularis, which causes dilatation of the membraneous and cartilaginous regions of the trachea and main bronchi. This increased wall compliance allows the development of large diverticular formations of musculo-membranous tissue redundant between the cartilaginous rings [13].

Some authors report that cases are sporadic [14] while others associate Mounier-Kuhn syndrome with familial susceptibility, probably through an autosomal recessive pattern in view of the association with Ehlers Danlos syndrome and *cutis laxa* in children [1,12]. Acquired forms have been described as a complication of pulmonary fibrosis in adults and mechanical ventilation in premature neonates [15].

Inhalation of chronic irritants present in cigarette smoke and air pollution may play an important role in the development of tracheobronchomegaly [13]. Our patient is considered a sporadic case since he does not have any of these risk factors for the Mounier-Kuhn syndrome.

Tracheal diverticula can be congenital or acquired. Most often congenital in origin, their pathogenesis is undetermined [16]. Acquired diverticula can occur at any level of the organ but are most common at the posterolateral wall, at the junction between the intrathoracic and extrathoracic portions of the trachea, which may result from an anatomical defect at this level [16], or from increased intraluminal pressure due to chronic coughing [17]. In our case, there was a diverticular formation in the anterior wall of the trachea observed on bronchoscopy.

Clinical signs are not specific. Patients often present with recurrent lower respiratory infections and a chronic cough. Occasionally, Mounier-Kuhn syndrome can be revealed by spontaneous pneumothorax, hemoptysis, or digital hippocrates [18].

Mounier-Kuhn syndrome can also include nasosinus polyposis and a polymalformative syndrome with bilateral ptosis, epicanthus, micrognatism, and excess upper lip skin [19]. This is the case of our patient who presented with recurrent respiratory infections with a history of hemoptysis and excess skin on the upper and lower lip.

The diagnosis of Mounier-Kuhn syndrome is based on well-codified measurements of the trachea and main bronchi. These measurements can be made on a chest x-ray but are much more precise on a chest CT [3,^20^,21]. It is defined by an increase in the transverse and sagittal diameter of the trachea beyond 25 and 27 mm, respectively, and/or an increase in the diameter of the right and left main bronchi beyond 18 and 21 mm in men. The same definition applies to women with measurements of 21, 23, 17.4, and 19.8 mm, respectively [22,23]. An increase in tracheal cross-sectional area beyond 371 mm² for men and 299 mm² for women also defines the disease [24]. For our patient, the tracheal cross-sectional diameter was 47.3 mm, the anteroposterior diameter was 26.7 mm, the left main bronchus diameter was 35.5 mm, and the right main bronchus diameter was 23.4 mm. Bronchoscopy remains a powerful diagnostic tool to detect dilatation of the trachea and main bronchi during inspiration and their constriction or even collapse during expiration and coughing [12]. This investigation showed in the case of our patient a significant dilatation of the trachea with a reduction of its diameter during expiration and coughing of more than 50%.

The most common pulmonary complications of Mounier-Kuhn syndrome are bronchiectasis, bullous emphysema, recurrent pneumonia, and aspergillosis [25]. Infection with atypical organisms, including tuberculous and nontuberculous mycobacteria, is a major problem that can complicate some cases [26]. Our patient had multiple sites of bronchial dilatation on chest CT (Fig. 4).

Lung function exploration may show an obstructive ventilatory disorder with increased total lung capacity and residual volume [27].

Treatment of symptomatic patients is based on respiratory physiotherapy and the use of antibiotics during infectious exacerbations [28]. Bronchodilators and corticosteroids are used to control symptoms [2]. Our patient had a moderate form of the disease. Symptomatic treatment was prescribed for the patient. He was treated with oxygen therapy as he desaturated at 91% in ambient air, respiratory physiotherapy for bronchial evacuation, systemic antibiotic therapy to treat the respiratory infection, inhaled corticosteroid therapy associated with a long-acting bronchodilator to improve the dyspnea which presents the patient, with favorable clinical evolution. Pneumococcal and influenza vaccines are recommended independently of age and symptomatology [29]. Smoking cessation is highly beneficial. Exposure to industrial and occupational irritants and pollutants should be minimized [30]. Bronchial dilation may require endoscopic treatment with a stent or surgical tracheobronchoplasty in severe cases [31].

## Authors’ contributions

A Imzil: writing the manuscript, patient care, radiological and bronchoscopic image processing, this author had read and approved the manuscript. F Bounoua : performing bronchoscopy, patient care, this author had read and approved the manuscript. H Naji Amrani: performing bronchoscopy, reviewed the paper, this author had read and approved the manuscript. H Moubachir: supervised and reviewed the paper, this author had read and approved the manuscript. H Serhane: diagnosis of the clinical case, supervised and reviewed the paper. this author had read and approved the manuscript.

## Patient consent statement

The authors of this manuscript have obtained written, informed consent from the patient to write up the case report and for the use of images pertinent to the case. We have ensured anonymity of all clinical and graphical data used.
